# Supplementary material for: MAX inactivation is an early event in GIST development that regulates p16 and cell proliferation
Source: Nat Commun. 2017 Mar 8;8:14674. doi: 10.1038/ncomms14674 (PMC5344969; doi:10.1038/ncomms14674)
Supplement: Supplementary Information — and Supplementary Tables [file ncomms14674-s1.pdf]

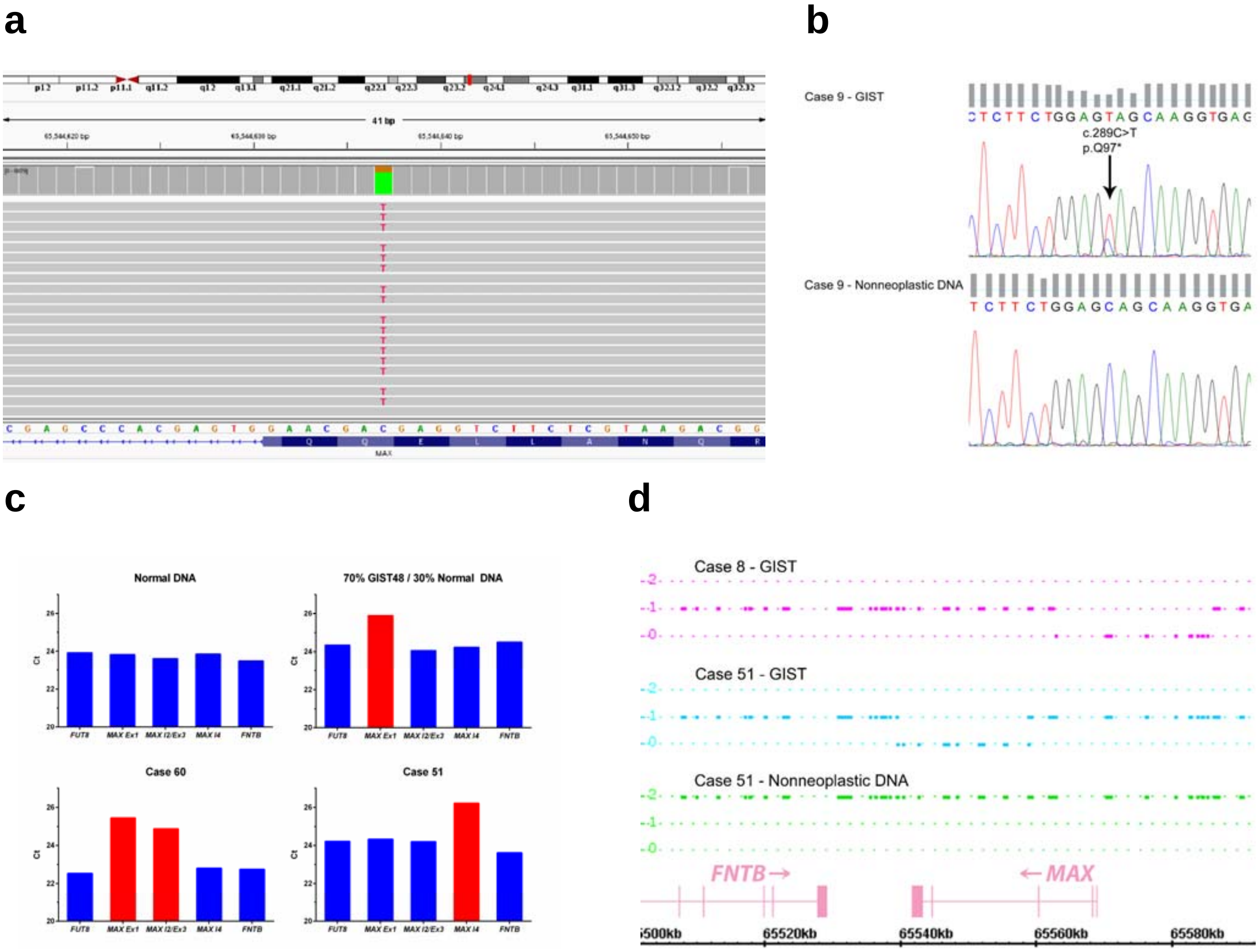

Supplementary Figure 1. Validation of *MAX* genomic inactivating mutations in GIST. A homozygous nonsense mutation (G>A; Q97*) in *MAX* exon 4, case 9, is detected by Ion AmpliSeq targeted sequencing (**a**) and validated by Sanger sequencing (**b**). Homozygous deletion of *MAX* exons 4-5 in case 51, and exons 1-3 in case 60, validated by quantitative PCR (**c**). A mixture of 70% mutant DNA from the *MAX*-mutant GIST48 cell line and 30% normal DNA served as control for calibration in order to model a typical percentage of 20-30% non-neoplastic cell DNA in GIST clinical biopsies. Homozygous deletion of *MAX* exons 1-2 in case 8 and exons 4-5 in case 51, validated by Cytoscan HD SNP arrays (**d**), showing case 8 (pink trace, top), and case 51 (blue trace, middle), compared to nonneoplastic DNA from case 51 (green trace, bottom).

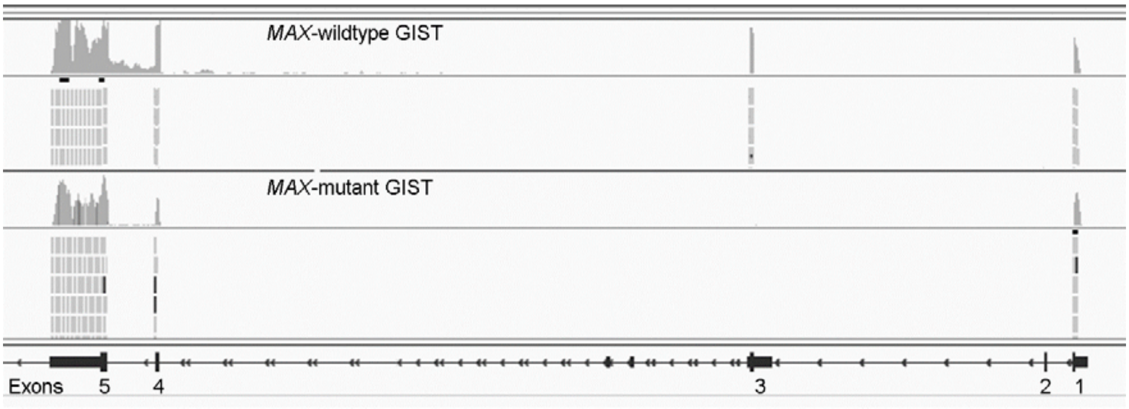

Supplementary Figure 2. Splice site mutation extinguishes expression of *MAX* exon 3. Genome-wide RNAseq demonstrates absence of *MAX* exon 3 transcripts in GIST case 7 (lower half of figure), due to a *MAX* splice site mutation. Control (upper half of figure) is a *MAX*-wildtype GIST. Transcripts were not assigned bioinformatically to exon 2, due to the extremely short (27nt) length of this exon.

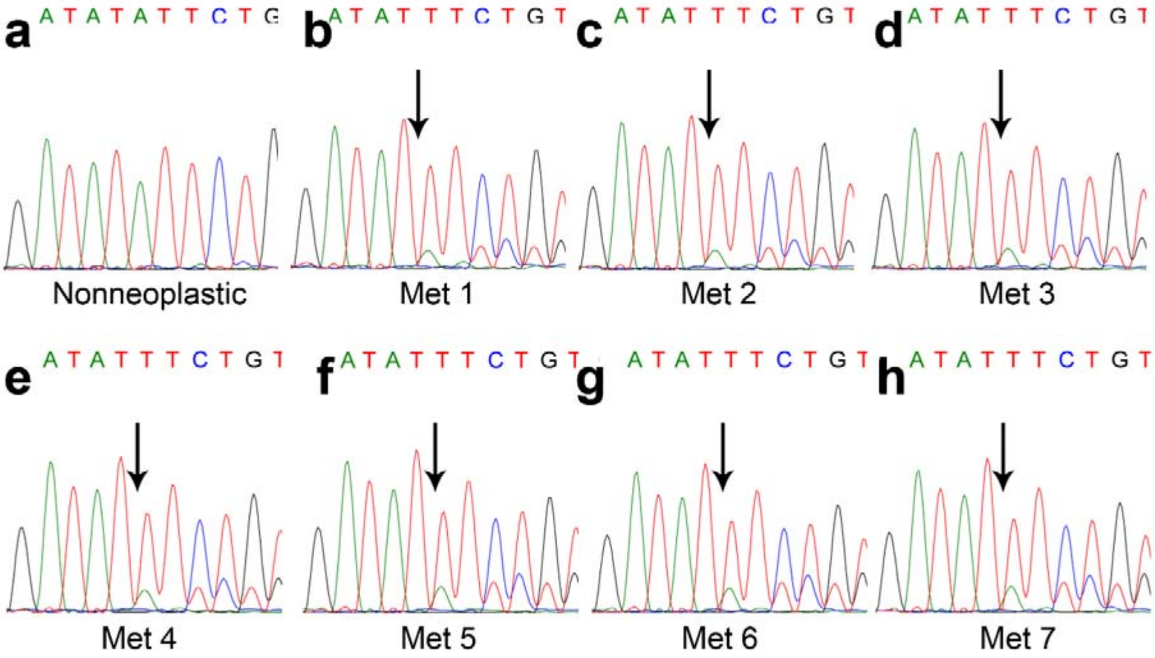

Supplementary Figure 3. Identical somatic *MAX* genomic inactivating mutations in GIST metastases from the same patient. Identical *MAX* frameshift mutations (Y70fsX169) are demonstrated in 7 GIST metastases from pt 53 (**b-h**) but not in nonneoplastic tissue from the patient (**a**).

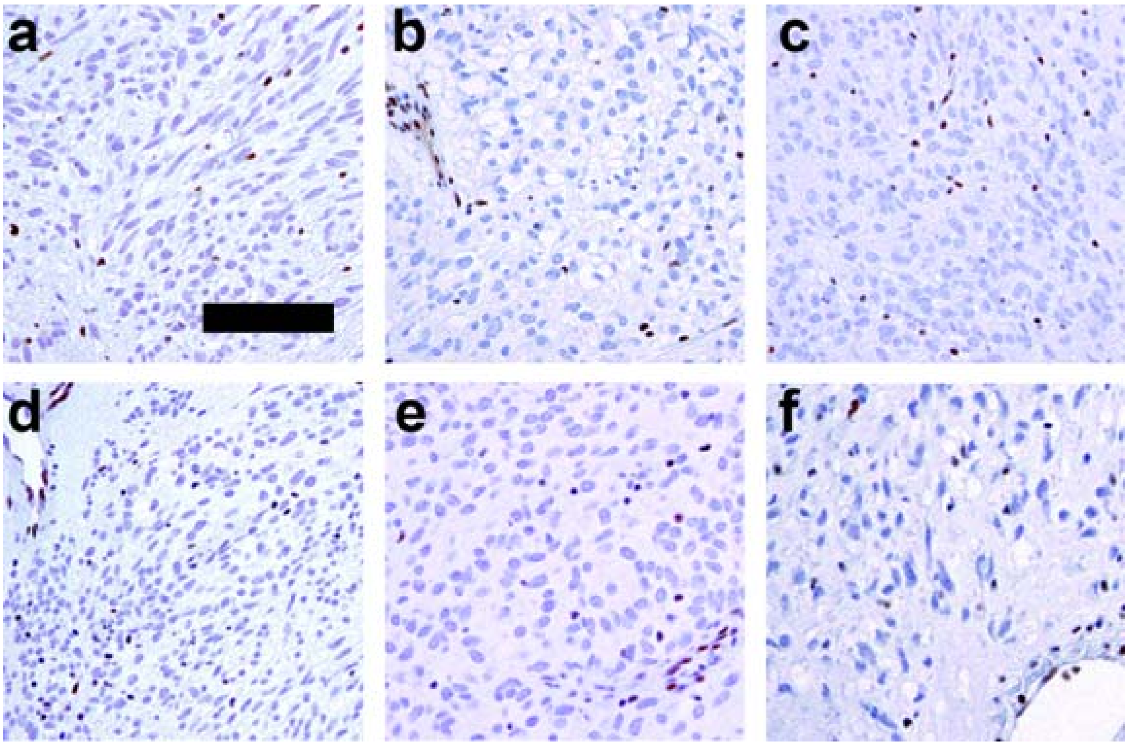

Supplementary Figure 4. MAX protein loss in GIST metastases from the same patient. Loss of MAX protein expression is demonstrated by immunohistochemistry in 6 GIST metastases (**a-f**) from pt 51 with homozygous deletion of *MAX* exons 4-5. Scale bar, 50 µm.

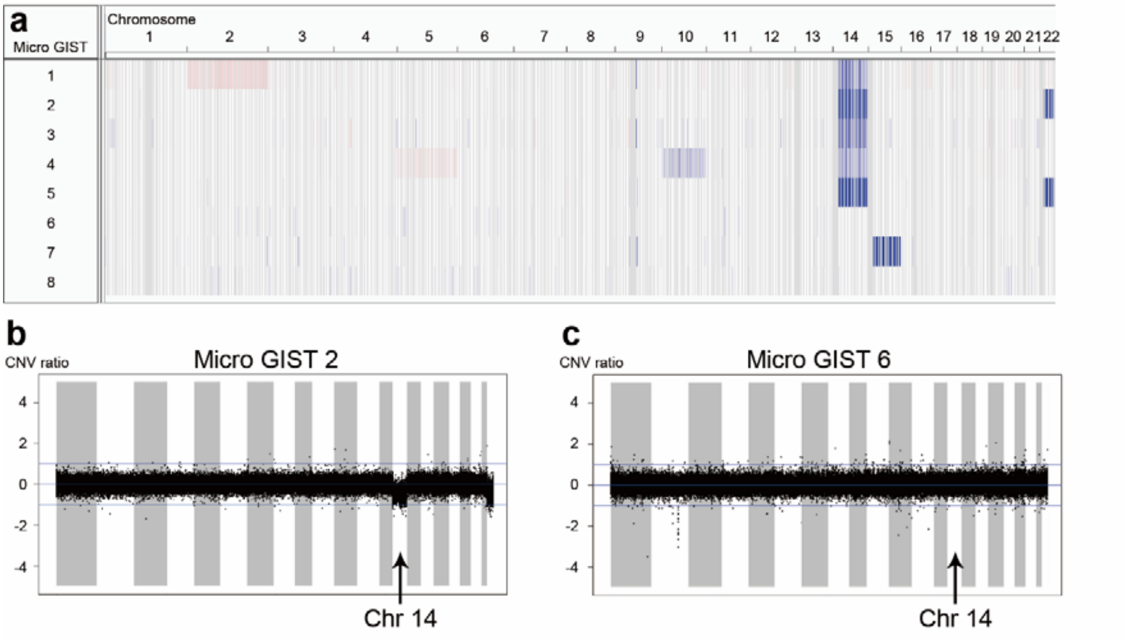

Supplementary Figure 5. Chromosome 14 deletions in micro GISTs. Chromosome 14 deletions are demonstrated by array CGH in 5 of 8 micro GISTs, which are the earliest identifiable stages of GIST development (**a**). Log2 ratio traces show 14q and 22q deletions in micro GIST 2 (**b**) compared to normal copy number in micro GIST 6 (**c**).

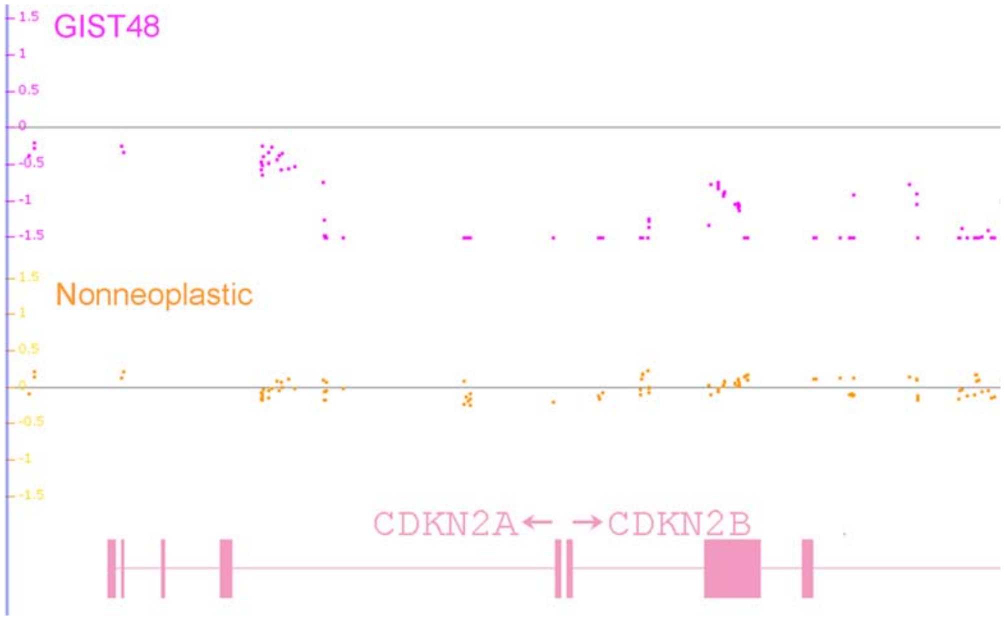

Supplementary Figure 6. *CDKN2A* deletion in GIST48 targets only p14ARF. Cytoscan HD SNP arrays for GIST48 (pink tracing) show complete deletion of *CDKN2A* exon 1, inactivating p14ARF but not p16INK4A. The nonneoplastic cell control is the orange tracing.

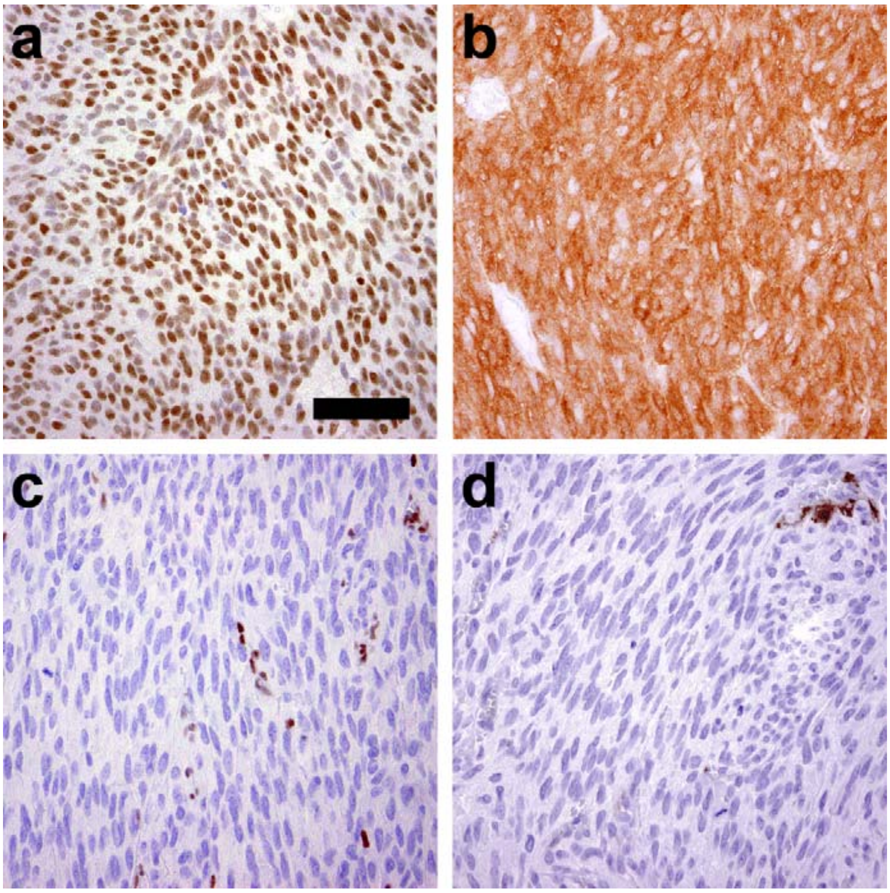

Supplementary Figure 7. MAX inactivation is associated with loss of p16 expression. Immunohistochemical evaluations show strong, diffuse expression of MAX (**a**) and p16 (**b**) in GIST case 67, which is wildtype for *MAX* and the p16 coding sequence. By contrast, both MAX (**c**) and p16 (**d**) expression are lost in GIST case 8, which has *MAX* intragenic homozygous deletion and is wildtype for the p16 coding sequence. Internal positive controls in (**c**) and (**d**) are nonneoplastic endothelial and inflammatory cells. Scale bar, 50 μm.

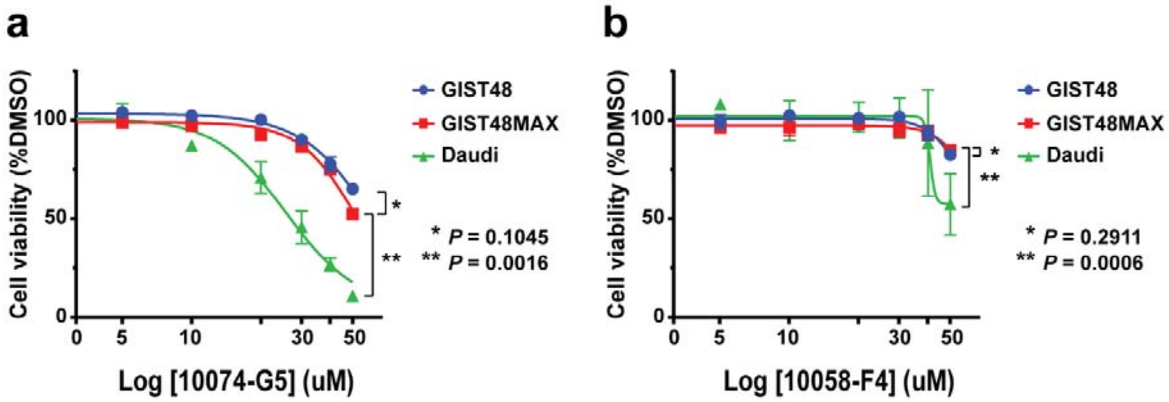

Supplementary Figure 8. MYC:MAX inhibitors do not have preferential activity in MAX-restored GIST. MYC-MAX heterodimerization inhibitors 10074-G5 (**a**) and 10058-F4 (**b**) do not have greater activity in GIST48 cells after MAX restoration (GIST48MAX = red curve) compared to parental MAX-deficient GIST48 (GIST48 = blue curve). MYC-dependent control cells are the Daudi Burkitt lymphoma line. Tests were performed in triplicates. Error bars show standard deviation.

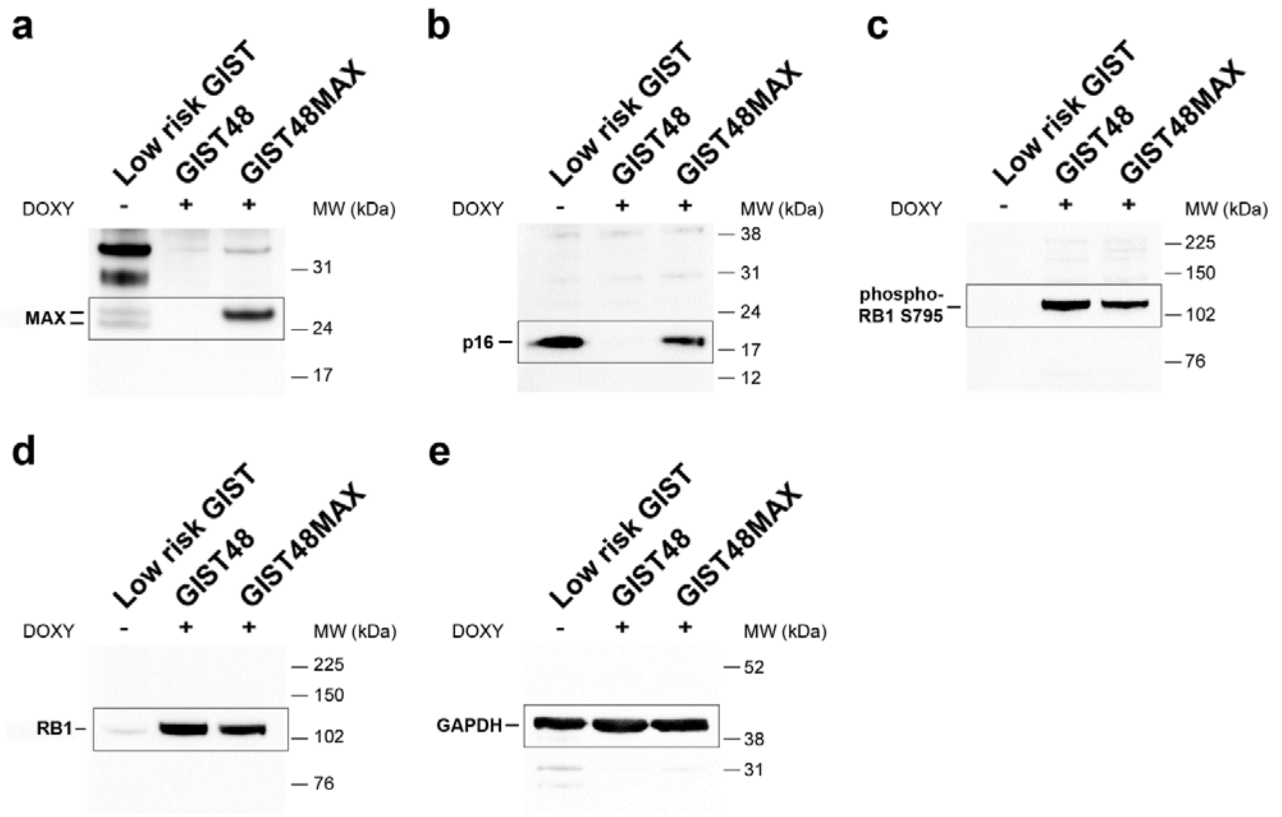

Supplementary Figure 9. MAX restoration also restores p16 expression and function in GIST. Uncropped immunoblots corresponding to Figure 5a: MAX restoration in *MAX*-mutant (homozygous deletion) GIST48 cell line restores p16 expression and inhibits CDK4/6-dependent phospho-RB1^{Ser795}; GAPDH serves as loading control. Black outlines delineate the fields used in the main text figure.

Supplementary Table 1. 14q and MAX IHC alterations in 8 micro GISTs.

Case ID	Site	Gender	Mutation	14q status (aCGH)	MAX IHC
1	Gastric	F	*PDGFRA*	Del	-
2	Gastric	F	*KIT*	Del	+
3	Gastric	M	*KIT*	Del	+
4	Gastric	M	*KIT*	Del	-
5	Gastric	M	*KIT*	Del	-
6	Gastric	M	*KIT*	Nl	+
7	Gastric	M	*KIT*	Nl	+
8	Gastric	F	*KIT*	Nl	+

Del: deletion; ex: exon; Nl: normal

Supplementary Table 2. Primers used for amplification and sequencing.

Gene	Exon	Designation	Sequence	Amplicon size [bp]	Application
MAX	1	E1+63F	TGTGAGTGAGAGAGCGAGTGA	387	Genomic PCR
MAX	1	I1+209R	ACTGGAGGTTGGGGGAAG		Genomic PCR
MAX	1	E1-365F	GGTTTCTTCCGGGATTTGTA	81	qPCR
MAX	1	E1-285R	AACTACAAGTCTCGGCTGCC		qPCR
MAX	2	I1-196F	GGACCCCTCTACCCTGCTAC	359	Genomic PCR
MAX	2	I2+136R	AACCACTAAGGTGAGGTGGG		Genomic PCR
MAX	3	I2-144F	CTAACTGCCCACCTCGAGAA	376	Genomic PCR
MAX	3	I3+124R	ATTCAGGTAGCATGGTTGCC		Genomic PCR
MAX	3	I2-129F	GAGAAATCCTTCCCAGTCCA	142	qPCR
MAX	3	E3+13R	CTTCTAGGCTGACAAACGGG		qPCR
MAX	4	I3-125F	TTCAGCCTTTTCCCACCTTA	372	Genomic PCR
MAX	4	I4+123R	AGGACCAAGCCTGCTACTGA		Genomic PCR
MAX	4	I4+145F	TGAGTGTTAGAGCCGTCCTG	70	qPCR
MAX	4	I4+214R	CCAACTTCCTAGCTTCCACTG		qPCR
MAX	5	I4-144F	CGAAGGGAGCTACTTCTGAGC	496	Genomic PCR
MAX	5	E5+352R	CGTAGAAGCTCTTGGACAACA		Genomic PCR
FNTB	1	E1-401F	TCTCGCTGAAATGAAATTCG	67	qPCR
FNTB	1	E1+44R	CTACGCTCTTGCAGCAGCT		qPCR
FUT8	1	E1-460F	CAGATTGCAACCTTGACAGG	140	qPCR
FUT8	1	E1-321R	AAATGCCCAAGCCTAGAGAA		qPCR
